# Comparison of Methods for Correction of Mortality Estimates for Loss to Follow-Up after ART Initiation: A Case of the Infectious Diseases Institute, Uganda

**DOI:** 10.1371/journal.pone.0083524

**Published:** 2013-12-31

**Authors:** Agnes N. Kiragga, Barbara Castelnuovo, Rachel Musomba, Jonathan Levin, Andrew Kambugu, Yukari C. Manabe, Constantin T. Yiannoutsos, Noah Kiwanuka

**Affiliations:** 1 Infectious Diseases Institute, College of Health Sciences, Makerere University, Kampala, Uganda; 2 Medical Research Council/Uganda Virus Research Institute Uganda Research Unit on AIDS, Entebbe, Uganda; 3 School of Medicine, Johns Hopkins University, Baltimore, Maryland, United States of America; 4 Indiana University Richard M. Fairbanks School of Public Health, Department of Biostatistics, Indianapolis, Indiana, United States of America; 5 School of Public Health, College of Health Sciences, Makerere University Kampala, Uganda; Technische Universität Dresden, Germany

## Abstract

**Background:**

In sub-Saharan Africa, a large proportion of HIV positive patients on antiretroviral therapy (ART) are lost to follow-up, some of whom are dead. The objective of this study was to validate methods used to correct mortality estimates for loss-to-follow-up using a cohort with complete death ascertainment.

**Methods:**

Routinely collected data from HIV patients initiating first line antiretroviral therapy (ART) at the Infectious Diseases Institute (IDI) (Routine Cohort) was used. Three methods to estimate mortality after initiation were: 1) standard Kaplan-Meier estimation (uncorrected method) that uses passively observed data; 2) double-sampling methods by Frangakis and Rubin (F&R) where deaths obtained from patient tracing studies are given a higher weight than those passively ascertained; 3) Nomogram proposed by Egger *et al*. Corrected mortality estimates in the Routine Cohort, were compared with the estimates from the IDI research observational cohort (Research Cohort), which was used as the “gold-standard”.

**Results:**

We included 5,633 patients from the Routine Cohort and 559 from the Research Cohort. Uncorrected mortality estimates (95% confidence interval [1]) in the Routine Cohort at 1, 2 and 3 years were 5.5% (4.9%–6.3%), 6.6% (5.9%–7.5%) and 7.4% (6.5%–8.5%), respectively. The F&R corrected estimates at 1, 2 and 3 years were 11.2% (5.8%–21.2%), 15.8% (9.9%–24.8%) and 18.5% (12.3% –27.2%) respectively. The estimates obtained from the Research Cohort were 15.6% (12.8%–18.9%), 17.5% (14.6%–21.0%) and 19.0% (15.3%–21.9%) at 1, 2 and 3 years respectively. Using the nomogram method in the Routine Cohort, the corrected programme-level mortality estimate in year 1 was 11.9% (8.0%–15.7%).

**Conclusion:**

Mortality adjustments provided by the F&R and nomogram methods are adequate and should be employed to correct mortality for loss-to-follow-up in large HIV care centres in Sub-Saharan Africa.

## Introduction

The scale-up of HIV care in resource limited settings, particularly in sub-Saharan Africa (SSA), has led to an increase in the number of centres where patients obtain combination antiretroviral therapy (ART) [2]. Previous studies have shown that about 80% of patients who initiate ART are retained in care at 12 months [3]. In addition, mortality rates are significantly higher among patients who drop out of care compared to those retained in care. Patients who drop out of care are often less adherent to ART medication, more immunosuppressed, and thus less likely to survive [4]. Estimates of survival will consequently be affected by the unavailability of data on patients who drop out of care. For program evaluations, it is therefore important to understand what happens to patients who drop out of care, in order to obtain less biased estimates of important HIV outcomes [5]–[8].

The traditional approach of estimating survival probabilities considers loss to follow-up as equivalent to administrative censoring at a subject's last visit date [4], [5]. This often leads to underestimation of cumulative mortality, since a considerable proportion of the patients who drop out of care may have subsequently died. Sample-based tracing is a recent methodology that involves tracing of a random sample of patients who are lost. A weight is generated as the inverse of the proportion of patients traced, out of all patients lost to follow-up. This method was proposed by Frangakis and Rubin, hereafter referred to as the F&R method [9]. In this method, the outcomes among patients who drop out of care and are not included in the random sample, are represented by up-weighting the outcomes among patients who were traced. This method has been applied to obtain less biased estimates of mortality among patients receiving ART in resource-limited settings, where the ‘true’ mortality estimate cannot be obtained due to the large proportions of patients who drop out of care, and for whom death or follow up status cannot be ascertained through death registries. Death registries are often absent in this SSA and may not be linked to patient data. Previous studies in South Africa, where death ascertainment is much more accurate because of the presence of death registries, and where unique patient identifiers enable patient linkage, also conclude that only reporting passively ascertained deaths often leads to significant underestimations of mortality estimates [1], [10].

In the absence of patient tracing, a nomogram approach can be used to obtain mortality estimates corrected for loss to follow-up [1]. The nomogram method proposed by Egger and colleagues generates a correction factor which is based on the percentage of patients lost to follow-up at specific time periods after ART initiation, and the estimated ratio of mortality between patients lost and patients not lost to follow-up [1]. The idea of the nomogram is based on an inverse relationship between the proportion of patients lost to follow-up and the mortality rate among these patients. It implicitly assumes that an increasing proportion of patients who disengage from care at one clinic will continue receiving care at another. With the rapid scale-up and increasing accessibility to care and treatment programs in sub-Saharan Africa, the defining hypothesis of the nomogram approach is plausible. As a result, the nomogram has generally resulted in reasonable adjustments of mortality estimates [1].

In the application of the nomogram method used to correct mortality rates in Tanzania, the year 1 unadjusted estimate was 9.5% and was improved to 15% [11]. Similarly, a recent study by Henriques and colleagues reported results from a comparison of methods used to correct mortality estimates in Malawi [4]. Methods used in this study included the F&R method (the presumed gold standard), the nomogram, a frailty-dependent stratified Kaplan Meier analysis, as well as survival regression analysis (based on observed data only). The authors showed that the unadjusted mortality estimates were lower than the corrected estimates that incorporated additional information from the patient tracing [4]. The nomogram method yielded intermediate results, with mortality rates that were slightly higher than the uncorrected mortality estimates after one year from ART initiation [4].

This study describes mortality estimates obtained from cohort of HIV infected persons receiving routine care and treatment at the Infectious Diseases Institute (IDI), hereafter referred to as the Routine Cohort. Nested within the IDI HIV clinic is an observational research cohort, hereafter referred to as the Research Cohort. There is limited loss to follow-up and complete outcome ascertainment in the Research Cohort. Consequently, the mortality estimates obtained from the Research Cohort are unaffected from unknown vital status among patients who drop out of care. We compared mortality estimates obtained after the application of methods for correcting mortality in the Routine cohort, to the estimates obtained from the Research Cohort. To our knowledge, this is the only paper in the literature which compares and validates the adjustments from a number of proposed methods for correction of mortality estimates for loss to follow-up, versus estimates derived from a cohort with negligible dropout, and thus free from such bias.

## Methods

### Ethics statement

This process was approved by the Institutional Review Board. The study and use of data was reviewed and approved by the Scientific Review Board of the Infectious Diseases Institute, the Institutional Review Board of Makerere University and the Uganda National Council for Science and Technology (HS683). Patients were not consented since data was primarily used for routine clinical care and no personal identifiers were made available to the researchers during the analysis.

### Study setting and population

The Infectious Diseases Institute (IDI) is a center of care and treatment, training and Research for HIV positive patients and is located in Kampala, the capital of Uganda. Currently, over 29,000 patients have been registered at IDI, and of these, about 7,000 are active in care and receiving antiretroviral therapy [12].

### The IDI Routine Cohort

Data collected during routine HIV care of patients are stored in the IDI clinic database. Details on the IDI clinic have previously been reported elsewhere [13]. In brief, patients have monthly clinical assessments while laboratory assessments such as complete blood counts (CBC) are performed every three months. CD4 T-cell count tests are performed every 6 months while HIV RNA viral load measurements are not routinely offered. All adult patients (18 years and above), registered at the IDI and initiated ART between 1^st^ January 2005 and 9^th^ December 2007 were included in this study. The date of database closure for the Routine Cohort was 9^th^ December 2007, and was the date of the first tracing exercise for all patients who had dropped out of care.

### The IDI Research Cohort

The IDI Research Cohort comprised of 559 patients who were enrolled between April 2004 and April 2005. All adult (18 years and above) patients were enrolled in the Research Cohort if they had been confirmed to have HIV, resided within a 20 Km radius of Kampala, were willing to be followed up for at least 2 years, were eligible for ART according to the WHO 2003 guidelines and Uganda Ministry of Health recommendations (CD4 cell count <200 cells/mL or WHO stage IV), and provided informed consent. In the Research Cohort, study visits take place every 3 months, in addition to the monthly visits in the Routine Cohort. Laboratory tests performed include CD4 T-cell counts by FACS Count (Becton Dickinson), HIV type 1 viral load measurements (every 6 months) with a detection limit of 400 copies/mL, and measurements of serum aspartate transaminase and creatinine levels performed every three months. Self reported adherence is measured at each of the study visits. Details of the Research Cohort procedures have been published previously [14], [15].

### Ascertainment of outcomes

All patients who missed any of their scheduled visits (3 months apart) in the Research Cohort were considered lost to follow-up, and were contacted by telephone after two weeks. Patients who were un-contactable on phone were traced at the home address given at study enrollment. Patients were asked to inform the study counselor if they decided to transfer to another HIV care facility, while patients' relatives were encouraged to inform the study counselor if the patient died. Consequently, the rate of loss to follow-up was very low and complete ascertainment of patient outcomes was achieved [15]. Therefore the mortality estimates obtained using the standard Kaplan Meier method in the Research Cohort were considered “true” estimates and were used to validate methods used in the Routine Cohort. In the Routine Cohort, patients were considered lost to follow-up if they had not returned for a clinic visit or drug pick-up in three months. At database closure, all patients considered lost to follow-up were contacted through telephone calls. In the event, that the person was un- contactable over the telephone, a home visit was arranged at the patient's home address.

### Statistical methods

Patients' characteristics at ART initiation in the Routine and Research cohorts were described using proportions, medians and interquartile ranges, while comparisons between groups were performed using χ^2^ tests for the categorical variables, and Mann-Whitney tests for the continuous variables. Baseline CD4 count was defined as the measurement taken at ART initiation for patients in Research Cohort. In the Routine Cohort however, the baseline CD4 count was any measurement taken within 3 months prior to ART initiation. When more than one CD4 count measurement was available, the one closest to the date of ART initiation was selected. Missing baseline CD4 counts were assumed to be missing at random and were imputed and replace with the average of measurements obtained from five rounds of multiple imputation. The multiple imputation was carried out using chained equations (ICE), where the distribution of the observed data which included age, gender, WHO clinical stage, baseline ART regimen was used to estimate a set of plausible values for the missing CD4 counts [16].

In both cohorts, survival time was estimated as the difference between the date of ART initiation and date of either loss to follow-up, death, self-transfer, last clinic visit or the date of database closure. Dates of death were confirmed from patients' relatives or next of kin, or from passively obtained information passed on to the clinic staff by representatives of the patients or HIV peer support group members. Among the patients who were successfully traced, all efforts were made to obtain a reasonably accurate date of death. If none was obtained, a date of death was imputed using information from similar patients who died who were matched according to baseline CD4 count and date of ART initiation. In the absence of baseline CD4 counts, similar patients who died were identified after matching according to WHO clinical stage.

The study used three methods to estimate mortality: Method 1 was the standard Kaplan-Meier (uncorrected method) estimation that uses passively observed data; Method 2 was the F&R method. While using this method, a weighted average of the hazards of death between the patients who were lost to follow-up and those retained in care was obtained [5], [17]. Method 3 was the nomogram by Egger and colleagues [1]. The nomogram graphically represents and plots the ratio of mortality among patients lost to follow-up and those not lost to follow-up, and a corresponding proportion of patients lost to lost follow-up. A correction factor is multiplied to the mortality observed among patients not lost to follow-up, in order to obtain corrected mortality ratios. There are two versions of the nomogram. The first, combines mortality estimates obtained from patients under observation and patients lost-to-follow-up and subsequently traced. The second version of the nomogram, uses a meta-regression method where correction factors are applied to observed mortality estimates based on the levels of lost-to-follow-up and coefficients determined from a meta-analysis of a number of ART programs in Sub-Saharan Africa [7]. The assumption of the second version of the nomogram does not use the additional data obtained from tracing studies, and assumes that the higher the proportion of loss-to-follow-up, the lower the mortality among patients who are no longer under observation. This method assumes that the high levels of loss-to-follow-up are due to increasing rates of patient self-referral to alternative care centers, in an environment of rapidly scale up of care and treatment. It is also important to note that this method is only used to estimate mortality within the first year of ART initiation. Our explicit objective was to assess, in our setting whether the nomogram method which is not dependent on the availability of additional information obtained from tracing, is adequate to provide reasonably accurate mortality estimates, in absence of tracing exercises.

We compared the estimates of mortality in the Routine Cohort that were obtained from these three methods, to those obtained from the Research cohort in the first three years from ART initiation. The mortality estimates from the Research Cohort were obtained using the standard Kaplan-Meier (uncorrected method) and were considered the “gold-standard” as they were likely unaffected by any biases resulting from losses from observation. We calculated differences between the mortality estimates obtained from both cohorts. All statistical analyses were performed using STATA 12.1, (Stata Corporation, College Station, Texas, USA).

## Results

### Baseline characteristics

Between 1^st^ January 2000 and 9^th^ December 2007, a total of 19562 patients were registered for care and treatment at the IDI clinic, and of these 6398 had been initiated on ART. Among the 6398, 765 had already initiated ART from other HIV care centres prior to registration, and vital information collected at ART initiation was not recorded. We excluded these 765 patients and remained with 5633 eligible for the analysis in the Routine Cohort. In the Research Cohort, during the period 1 April 2004 to 30 April 2005, a total of 1792 patients were eligible for the ART and of these 559 were randomly selected and prospectively enrolled. Generally, patients in both cohorts had similar baseline characteristics (see Table 1). In both cohorts, CD4 count at ART initiation was low with median (IQR) 93 cells/μl [28, 164] and 93 cells/μl [21, 166] in the Research and Routine cohorts respectively. In the Research Cohort, only 3 patients had a missing CD4 count at ART initiation while 1658 (29.4%) of the patients had missing CD4 count at ART initiation in the Routine Cohort. Patients in the Research Cohort returned to the clinic every 3 months, and the average (standard deviation) number of visits was 9 (4). In the Routine Cohort, the patients were seen monthly, and the average number of visits was 31(19). The mean percentage adherence to ART in both cohorts was 98%.

**Table 1:** Baseline characteristics for Routine and Research cohorts.

Variable	Routine cohort (n = 5633)	Research cohort (n = 559)	P -value
Male sex, n; (%)	2018 (36)	173 (31%)	0.021
Age, years (median; IQR)	36 (31–42)	38 (33–44)	0.033
Pre-therapy CD4 cell count^§^	93 (28–164)	98 (21–163)	0.448
(cells/μL; median; IQR)			
WHO stage III/IV, n (%)	3991 (71)	496 (89)	<0.001
Pre-therapy BMI (Kg/m^2^; median; IQR)	21 (19–23)	20 (17–22)	0.035
ART Regimen, n (%)			
d4T +3TC + Nevirapine	3520 (62.5)	413 (73.9)	<0.001
AZT +3TC + Efavirenz	2050 (36.4)	144 (25.7)	
Other 1^st^ line regimen	63 (1.1)	2 (0.4)	
Date of ART initiation (median; IQR)	January 24, 2006 (May 13, 2005– February 6, 2007	January 17, 2005 (November 24, 2004– April 11, 2005)	<0.001

ART  =  Antiretroviral therapy, WHO World Health Organization, d4T  =  stavudine, AZT  =  Zidovudine, 3TC  =  lamivudine, IQR  =  Interquartile range, BMI Body Mass Index ^§^ 3 patients from Research cohort and 1658 from the Routine cohort had missing Pre-ART CD4 counts.

### Loss to follow-up

In the Research Cohort, 20 (3.5%) patients were lost to follow-up and a total of 99 deaths were observed during the study period. A date of death was recorded for all Research Cohort patients who had died. In the Routine Cohort a total of 806 (14.3%) patients were lost to follow-up and of these, 406 (50.4%) with contact information were traced. Among the patients traced, 107 (26.3%) were dead, 69 (17.0%) were self-transferred to another HIV care center, while 230 (56.7%) could not be located. The total number of deaths in the Routine Cohort was 469 (362+107 deaths obtained passively and from tracing, respectively). A total of 231 (49%) patients had missing dates of death. Table 2 shows the characteristics of three groups of patients in the Routine Cohort; patients lost and not traced due to lack of contact information (n = 400), patients lost traced and found (n = 176), and patients lost, traced and not found (n = 230). When we compared the group of patients who were lost, traced and found with the patients who were lost and not traced, we observed that the former had a slightly higher proportion of the patients with advanced disease (WHO clinical stage III–IV) (80.7% versus 73.0%, P = 0.05), a higher proportion were males (46.6% versus 37.0%, P = 0.034) compared to the latter. Patients who were not found during the tracing exercise had similar pre-ART CD4 cell counts, Body Mass Index (BMI) and duration on ART compared to patients who were not traced. Generally, patients who initiated ART earlier were less likely to be found during the tracing exercise (Table 2).

**Table 2:** Differences between patients lost and not traced, patients lost, traced and found and patients lost, traced and not found in the Routine Cohort.

Variable	Lost, not traced (N = 400)	Lost, traced and found (N = 176)	Lost, traced not found (N = 230)
Male sex, n (%)	148 (37.0)	82 (46.6)	86 (37.4)
Age, years (median; IQR)	36 [31, 42]	37 [32, 44]	36 [31, 41]
WHO stage III/IV, n (%)	292 (73.0)	142 (80.7)	180 (78.3)
Pre-therapy BMI			
(Kg/m^2^; median; IQR)	20.5 [18.0, 23.5]	21.0 [18.3, 23.0]	20.1 [17.8, 22.4]
ART Regimen, n (%)			
d4T +3TC + Nevirapine	252 (63.0)	118 (67.0)	179 (77.8)
AZT +3TC + Efavirenz	137 (34.2)	52 (29.6)	49 (21.3)
Other 1^st^ line regimen	11 (2.8)	6 (3.4)	2 (0.9)
Date of ART initiation, median, (IQR)		06/27/2006 [09/30/2005−03/19/2007]	11/30/2005 [05/27/2005–10/30/2006]
Duration of ART before loss to follow-up, median (IQR) days	63 [9, 245]	74 [14, 294]	87 [16, 255]
Pre-therapy CD4 cell count (cells/μL), median (IQR)	91 [15, 190]	67 [12, 173]	78 [16, 161]
Duration lost prior to tracing, (days) median (IQR)	N/A	278 [126, 549]	496 [241, 774]

ART  =  Antiretroviral therapy, WHO World Health Organization, d4T  =  stavudine, AZT  =  Zidovudine, 3TC  =  lamivudine, IQR  =  Interquartile range, BMI Body Mass Index.

### Naïve and F&R-adjusted mortality estimates

**Figure 1:**
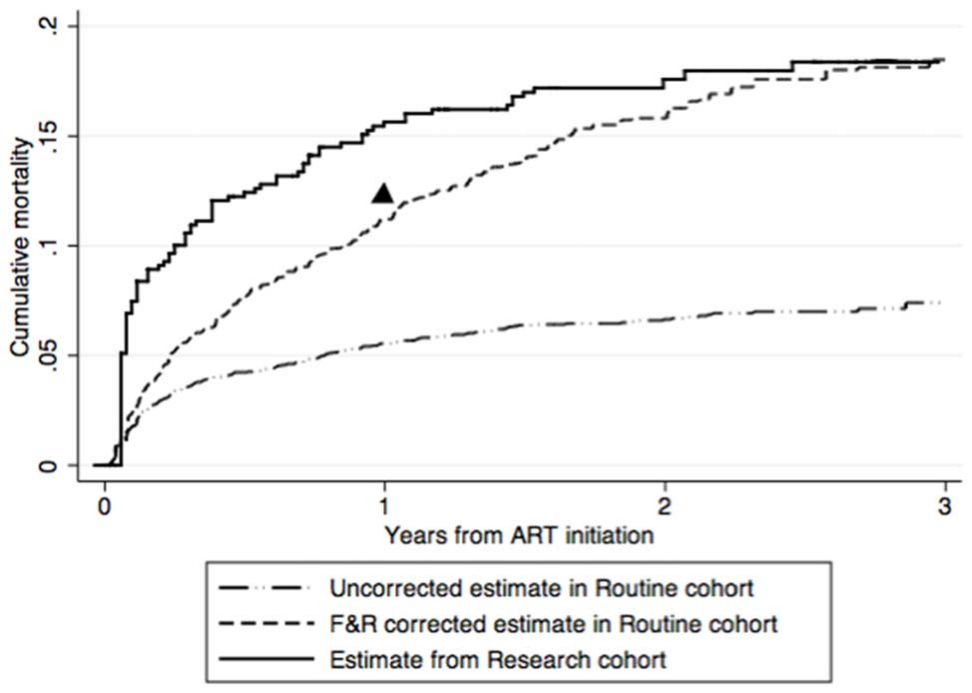
Uncorrected and F&R corrected cumulative mortality estimates in Routine and Research cohorts. The black triangle relates to the 1^st^ year mortality estimate obtained from the meta -analytic version of the nomogram method.

The uncorrected mortality estimates (95% confidence interval – CI) in the Routine Cohort after 1, 2 and 3 years from ART initiation were 5.6% (95% CI 4.9%–6.3%), 6.6% (95% CI 5.9%–7.5%) and 7.4% (95% CI 6.5%–8.5%) respectively. Using the F&R method, the 176 (69+107) patients who were initially lost to follow-up and were successfully traced, out of the 806 patients who were lost to follow-up, received a weight of 4.57 (806/176). This means that each successfully traced patient represented, in addition to himself or herself, 4.57 patients lost to follow-up and not traced. In this manner, corrections to the mortality estimates were obtained. The corrected estimates were 11.2% (95% CI 5.8%–21.2%), 15.8% (95% CI 9.9%–24.8%) and 18.5% (95% CI 12.3%–27.2%) at 1, 2 and 3 years after ART initiation. The mortality estimates obtained from the Research Cohort for the 1^st^, 2^nd^ and 3^rd^ year after ART initiation were 15.6% (95% CI 12.8%–18.9%), 17.5% (95% CI 14.6%–21.0%) and 19.0% (95% CI 15.3%–21.9%) respectively (See figure 1). The differences between the estimates obtained from the F&R method in the Routine Cohort and the Research Cohort mortality estimates were 4.4%, 1.7% and 0.5% after 1, 2 and 3 from ART initiation (Table 3).

**Table 3:** Comparison on methods for correcting mortality in the Research and Routine cohorts.

Variable	Research cohort, (n = 5633)	Routine cohort, (n = 559)					
	Standard Kaplan Meier method	Uncorrected (naïve) estimates	F & R method	Nomogram^a^ (meta-analytic method)	Difference between uncorrected and estimates from Research cohort	Difference between F&R and estimates from Research cohort	Difference between nomogram and estimates from Research cohort
Year							
1	15.6 [12.8, 18.9]	5.6 [4.9, 6.3]	11.2 [5.8, 21.2]	11.9 [8.0, 15.7]	10.0%	4.4%	3.7%
2	17.5 [14.6, 21.0]	6.6 [5.9, 7.5]	15.8 [9.9, 24.8]		10.9%	1.7%	
3	19.0 [15.3, 21.9]	7.4 [6.5, 8.5]	18.5 [12.3, 27.2]		11.6%	0.5%	

**a** The corrected mortality estimates with 95% CIs were obtained from the web calculator available at http://www.iedea-sa.org.

A comparison of the F&R corrected estimates in the Routine Cohort and uncorrected (“true”) estimates in the Research Cohort by baseline CD4 cell count, showed that differences between the two estimates were largest among patients with the lowest CD4 counts (CD4<50 cells/μl), 17.5% versus 23.7%, followed by the next CD4 strata (CD4 50–100 cells/μl) 8.7% versus 13.5%, and 6.5% versus 9.5% among patients with CD4 ≥100 cells/μl.

### The nomogram corrected estimate

Using the meta-regression method of the nomogram, the corrected mortality estimate in the Routine Cohort after the first year of ART initiation was 11.9% (95% CI 8.0% –15%.), as obtained from the web calculator [18]. The mortality estimates were 3.7% lower than the mortality estimates from the Research Cohort in the first year (see figure 1).

### Sensitivity analyses

A sensitivity analysis was performed to include only patients in the Routine Cohort who initiated ART between April 2004 and April 2005. The results showed very slight differences from the former estimates obtained using the F&R method. The mortality estimates from this sensitivity analysis were 6.6% (95% CI 3.2%–13.3%), 9.5% (95% CI 5.5%–15.9%) and 11.3% (95% CI 7.2%–17.6%) in years 1, 2 and 3 respectively. In another sensitivity analysis, we report mortality estimates obtained when the analysis was restricted to patients in the Routine Cohort with advanced disease (WHO stage III–IV). The mortality estimates obtained moved closer to those obtained from the Research Cohort, with 13.6% (95% CI 6.8%–26.3%), 18.2% (95% CI 10.9%–29.7%) and 21.0% (95% CI 13.4%–31.9%) in years 1, 2 and 3 respectively.

## Discussion

To our knowledge, this is the only paper in the literature, which compares mathematically adjusted mortality estimates to those derived from a cohort without biases emanating from loss to follow-up. In our setting we found that among patients lost to follow-up who were successfully traced, 28.3% had in fact died. This proportion is comparable to estimates reported in South Africa where 28.8% of the identifiable patients who were lost to follow-up had died within the first three months after ART initiation [10]. The year 1 mortality estimate obtained using the F&R method in the Routine Cohort was 11.2% (95% CI 5.8%–21.2%), and this was closer to the estimate from the Research Cohort, 15.6% (95%CI 12.8%–18.9%). The estimate from the Routine Cohort was within the 95% confidence intervals of the Research Cohort estimates. The proximity of the F&R adjustments was even closer in the second and third years after ART initiation. It is important to note that a lower proportion, (71%) of patients in the Routine Cohort had advanced clinical stage (WHO stage III–IV) compared with 89% in the Research cohort (Table 1). In an analysis restricted to patients with advanced disease (clinical stage WHO III–IV) in the Routine Cohort, the estimates changed appreciably and moved much closer to those in the Research Cohort. The lower mortality in the Routine Cohort could be explained by the immune status of the patients and largely due to patient drop out.

Previous studies report adjusted mortality estimates obtained after sample-based tracing in Uganda and Kenya [8] [17] [5]. In South Western Uganda where sample-based tracing was carried out, the F&R corrected incidence of death among patients that were lost to follow-up was 9.1% (95% CI 5.0%−16.0%) in the first year of ART initiation [8]. In Eldoret Kenya, where census-based tracing of all patients lost to follow-up occurred, the mortality estimate after the first year of ART initiation using the F&R method was 10.7% (95% CI 8.9% –12.6%), compared with the uncorrected estimate of 3.4% (95% CI 2.9%–4.0%) [17]. Using the F&R method with results from either sample or census based tracing exercise significantly increased the estimated mortality. Thus, results from tracing studies should be made available to the scientific community to enable adjusted estimates in similar regions to benefit programs that cannot afford to actively trace patients [1].

Application of the nomogram significantly increased estimates of mortality after ART initiation over the unadjusted mortality estimates. In our study, the nomogram improved the estimates from the Routine cohort from 5.5% to 11.9% in the first year, thus getting closer to estimates from the Research cohort. In Tanzania, the nomogram improved the cumulative probability of death from 9.4% to 15% [11]. Similarly in Kenya, the nomogram yielded a correction factor of 4.3, which led to a corrected estimate of 9.5% [1]. However, highlighted by Egger et al, the meta-analytic version of the nomogram cannot be used to estimate mortality among patients lost to follow-up after the first year of ART initiation, and such maybe limited in use. The nomogram relies of a major assumption that patients who drop out of care are able to obtain care from other facilities. In fact, in our study, this was assumption was achieved, since the IDI is situated in Kampala city, which is surrounded by several other ART service providers. Therefore patients who dropped out of care in our institution could have received care from another HIV care facility.

In general, we note that the number of ART service providers is generally increasing Sub-Saharan Africa [2], [19]. This has been reported in South Western Uganda where the number of ART care providers increased and 83% (95% CI 70%–93%) of who had dropped out of care had received care and treatment at another facility [20]. Despite these noted increases ART care providers, the assumptions of the nomogram may be overly strong in countries with few clinics.

In addition, under certain circumstances, the results from the nomogram may be much lower than those obtained using the F&R method. This appeared to be the case in the study by Henriques and colleagues [4] where the mortality adjustment from the nomogram was much lower than the estimate from the F&R method. This could be have been attributed to the low (8.7%) rate of loss to follow-up in the first year, and the vital status among those lost was assessed in a relatively small (34.6%) proportion of patients. In our study, the overall rate of loss to follow-up was slightly higher (14.3%) and the vital status was assessed among an even smaller (21.2%) proportion of the patients. Such factors could affect the performance of the nomogram.

From our experience, it is very important to differentiate passive information from information obtained from tracing studies. Information is passive if obtained as part of routine clinic updates during patients' monthly clinical assessments. Such information is passed on to clinic staff, and updated in the routine clinical database. On the other hand, information obtained through tracing studies is not passive and should be coded differently in the routine clinical database. Failure to distinguish passive information and that obtained from tracing studies may lead to an underestimation of the F&R weights, which could eventually generate lower mortality adjustments.

There were significant limitations to our analysis. Obviously, all our conclusions are predicated on the assumption that patients within the Research Cohort and Routine Cohort are similar in critical aspects of their disease prognosis. This is reasonable since the Research Cohort is comprised of patients from the same catchment area (Kampala) recruited by the same site (IDI). The difference in the clinical status of the patients in the Routine Cohort and the Research Cohort could have contributed to the difference in corrected mortality estimates. This is because patients in the Research cohort initiated ART at the very start of ART rollout in the region, whereas those in the Routine cohort were less sick over time [13]. This is likely since sensitivity analyses focusing on Routine cohort patients with advanced disease and Research cohort patients showed differences from the overall results.

A more significant limitation relates to the fact that during the implementation of the patient outreach at the IDI, the patients traced were not randomly selected, but were selected rather as a result of an administrative directive, geared towards ascertaining the outcomes of patients who were not returning for their scheduled appointments. This resulted in patients who were lost more recently to the study to be traced at higher rates compared to patients who had enrolled or started therapy earlier, contravening a central assumption of the methodology. It is reassuring however that, with the possible exception of the first year mortality estimate, the F&R-adjusted estimates for years 2 and 3 were extremely close to those obtained from analysis of the Routine cohort.

The F&R method has been implemented by a number of studies and has invariably resulted in significant upward corrections of unadjusted mortality estimates. A recent study by Schomaker et al, suggests additional methods of improving the estimates from the F&R method [21]. The method suggests the use two stage Inverse probability weighting through the use of logistic regression where the differences between the patients lost and not traced, and those lost, traced and found are reflected in weights. This approach would probably lead to better estimates of the F&R method.

In table 2 we compared a number of characteristics across the three groups of patients. There were no noted differences between indicators of health status in the three groups, and, also important to note that the patients who were not traced had the lowest proportion (73% versus 80.7% and 78.3%) of patients with WHO clinical stage III–IV. This could imply that they were not as sick and maybe the mortality among this group would be comparable with that obtained among the patients who were found during the tracing. Due to similar patient characteristics among patients in the three groups, no major changes in the weights given the available data were expected, and the observed differences would not necessarily affect the mortality estimates obtained from F&R method.

As such, this is the first attempt at validating the adjustments produced by a number of methods proposed in the literature. Many tracing studies have been carried out in a cross sectional manner, however, it is important to develop new methods that account for the fact that tracing is an on going activity, and adjustments should be made to account for this in the corrected mortality estimates.
